# AD Resemblance Atrophy Index of Brain Magnetic Resonance Imaging in Predicting the Progression of Mild Cognitive Impairment Carrying Apolipoprotein E-ε4 Allele

**DOI:** 10.3389/fnagi.2022.859492

**Published:** 2022-04-28

**Authors:** Yingren Mai, Zhiyu Cao, Jiaxin Xu, Qun Yu, Shaoqing Yang, Jingyi Tang, Lei Zhao, Wenli Fang, Yishan Luo, Ming Lei, Vincent C. T. Mok, Lin Shi, Wang Liao, Jun Liu, the Alzheimer’s Disease Neuroimaging Initiative

**Affiliations:** ^1^Department of Neurology, Sun Yat-sen Memorial Hospital, Sun Yat-sen University, Guangzhou, China; ^2^Department of Neurology, The Second Affiliated Hospital of Guangzhou Medical University, Guangzhou, China; ^3^BrainNow Research Institute, Shenzhen, China; ^4^BrainNow Medical Technology Limited, Shenzhen, China; ^5^Division of Neurology, Department of Medicine and Therapeutics, Gerald Choa Neuroscience Centre, Lui Che Woo Institute of Innovative Medicine, The Chinese University of Hong Kong, Shatin, Hong Kong SAR, China; ^6^Department of Imaging and Interventional Radiology, The Chinese University of Hong Kong, Shatin, Hong Kong SAR, China

**Keywords:** Alzheimer’s disease, mild cognitive impairment, magnetic resonance imaging, AD resemblance atrophy index, APOE ε4-allele, cognition

## Abstract

**Background and Objective:**

Early identification is important for timely Alzheimer’s disease (AD) treatment. Apolipoprotein E ε4 allele (APOE-ε4) is an important genetic risk factor for sporadic AD. The AD-Resemblance Atrophy Index (RAI)—a structural magnetic resonance imaging-derived composite index—was found to predict the risk of progression from mild cognitive impairment (MCI) to AD. Therefore, we investigated whether the AD-RAI can predict cognitive decline and progression to AD in patients with MCI carrying APOE ε4.

**Methods:**

We included 733 participants with MCI from the Alzheimer’s Disease Neuroimaging Initiative Database (ADNI). Their APOE genotypes, cognitive performance, and levels of AD-RAI were assessed at baseline and follow-up. Linear regression models were used to test the correlations between the AD-RAI and baseline cognitive measures, and linear mixed models with random intercepts and slopes were applied to investigate whether AD-RAI and APOE-ε4 can predict the level of cognitive decline. Cox proportional risk regression models were used to test the association of AD-RAI and APOE status with the progression from MCI to AD.

**Results:**

The baseline AD-RAI was higher in the MCI converted to AD group than in the MCI stable group (*P* < 0.001). The AD-RAI was significantly correlated with cognition, and had a synergistic effect with APOE-ε4 to predict the rate of cognitive decline. The AD-RAI predicted the risk and timing of MCI progression to AD. Based on the MCI population carrying APOE-ε4, the median time to progression from MCI to AD was 24 months if the AD-RAI > 0.5, while the median time to progression from MCI to AD was 96 months for patients with an AD-RAI ≤ 0.5.

**Conclusion:**

The AD-RAI can predict the risk of progression to AD in people with MCI carrying APOE ε4, is strongly correlated with cognition, and can predict cognitive decline.

## Introduction

With more than 55 million people suffering from dementia worldwide in 2021, the prevalence of dementia has increased dramatically, and is now the most common disease among older people (Scheltens et al., 2021; The Alzheimer’s Association, 2021). Alzheimer’s disease (AD) is the most common type of dementia, with sporadic AD being the most common. However, the pathogenesis and etiology of AD are not yet fully understood, and treatment options and efficacy are unsatisfactory (Hodson, 2018; Liu et al., 2018; Scheltens et al., 2021). The preclinical stage of AD is mild cognitive impairment (MCI), which mainly manifests as amnesia with the preservation of other cognitive and life skills, but MCI is often considered as a sign of normal aging (Petersen, 2011; Petersen et al., 2014). The early stage of AD is the best time for patients to begin treatment (Sperling et al., 2011), and clinical or community screening to identify MCI is therefore an important tool in AD treatment.

The apolipoprotein E ε4 allele (APOE-ε4) is a key gene associated with sporadic AD, and is one of the major risk factors for AD (Corder et al., 1993; Strittmatter et al., 1993; Genin et al., 2011; Verghese et al., 2011). APOE-ε4 promotes the decrease of β-amyloid (Aβ) degradation and the brain metabolic dysfunction; this exacerbates Aβ plaque formation and tau protein phosphorylation, which in turn accelerates hippocampal atrophy (Jiang et al., 2008; Liu et al., 2015; Zalocusky et al., 2019). The risk of AD is up to 10–60 fold higher in APOE-ε4 homozygous carriers, but the ε4 allele can not identify the onset of AD or predict the rate at which MCI progresses to AD (Farrer et al., 1997). Early identification of people with MCI carrying APOE-ε4 who may progress to AD is important for the aging community and within clinics.

The pathological changes in AD, mainly Aβ deposition, tau protein phosphorylation, and neuronal necrosis, which lead to brain atrophy, can begin decades before the onset of clinical symptoms in patient with MCI (Hardy and Higgins, 1992; Dubois et al., 2021). The diagnostic methods of AD and MCI in terms of pathological examinations include cerebrospinal fluid (CSF) examination and positron emission tomography-computed tomography, but their high cost, invasiveness, and the difficulty in obtaining detection reagents make them unsuitable for routine clinical examination (Molinuevo et al., 2018; Wei et al., 2019). Magnetic resonance imaging (MRI) is a widely used clinical tool to diagnose AD and MCI. MRI is non-invasive, non-radioactive, easy to obtain, inexpensive, and can be used to monitor the progression of AD (Frisoni et al., 2010; Knight et al., 2016). With the development of neuroimaging machine learning, many studies have found that a single MRI metric can predict the risk of MCI progression to AD. For instance, hippocampal atrophy was found to predict the onset of AD in healthy populations decades in advance (Den Heijer et al., 2010). The AD-Resemblance Atrophy Index (AD-RAI) is a whole-brain model-based MRI machine-learning-derived index reflecting the similarity of an individual’s atrophy pattern with that of AD patients, and is metric. Our recent study showed that the AD-RAI could accurately distinguish healthy individuals from patients with AD and predict the progression to MCI and AD with a higher accuracy than using a single brain structure (Zhao et al., 2019; Liu et al., 2021; Mai et al., 2021).

In this study, we explored whether the AD-RAI imaging index predicts progression to AD in patients with MCI carrying the APOE ε4 allele. We also analyzed the association between the AD-RAI and cognitive functioning, and whether the AD-RAI predicts cognitive decline.

## Materials and Methods

### Subjects

We used data from study subjects of the multicenter study Alzheimer’s Disease Neuroimaging Initiative Database (ADNI), a longitudinal multicenter study established in 2003 with the primary goal of investigating whether MRI, positron emission tomography-computed tomography, other biological markers, and clinical and neuropsychological assessments can be used to measure the progression of MCI and early AD (Mueller et al., 2005). The use of all data was approved by the Institutional Review Board of the ADNI website, and all patients signed an informed consent form. All relevant tests and methods were performed in accordance with relevant guidelines and regulations, and this study was approved by the ADNI Publications Committee.

Data on baseline demographic characteristics, APOE alleles, longitudinal neuropsychological cognitive assessments, and head MRI were collected. Only 733 patients with MCI from the ADNI-1, ADNII-GO/2, and ADNI-3 were included in this study, and detailed inclusion and exclusion criteria can be found on the ADNI website.^1^ Briefly, the inclusion criteria in this study were as follows: The diagnosis of MCI was made at first admission (see diagnostic table for details: DXSUM_PDXCONV_ADNIALL), and patients had a baseline Mini-Mental State Examination (MMSE) scores of 24–30; patients experienced subjective memory decline, as reported by study subjects, informants, and/or clinicians, and objective memory decline was measured by delayed memory scores (scores adjusted by years of education) on the Wechsler Memory Scale; patients had no impairment in other cognitive domains, basic preservation of the ability to perform daily tasks, and the absence of dementia. For the present study, we included patients with MCI who were followed up with diagnostic and neuropsychological assessments for a mean follow-up period of 36 months, with the diagnostic follow-up ranging from 12 to 180 months and the cognitive assessment follow-up ranging from 12 to 192 months. Subjects with MCI were divided into two groups according to their diagnosis during the follow-up period, as follows: subjects with clinical symptoms and cognitive levels that met the diagnostic criteria for likely AD (2011 NIA-AA diagnostic criteria or other AD diagnostic criteria) (Mckhann et al., 2011) during the follow-up period were allocated to the MCI conversion group (MCIc group), while those with MCI who did not convert to normal functioning or to AD at the end of the follow-up period were allocated to the stable MCI group (MCIs group).

### Neuropsychological Assessments

The cognitive measures included in our study were the MMSE, Clinical Dementia Rating Scale Sum of Boxes (CDR-SOB), composite scores for memory (ADNI-MEM), and executive functions (ADNI-EF). The ADNI-MEM assessment is a comprehensive tool that integrates all memory items, and its composite z score is based on the three-word recall item from the MMSE, recognition tasks from The Alzheimer’s Disease Assessment Scale–Cognitive Subscale (ADAS-Cog), recall from Logical Memory I of the Wechsler Memory Test-Revised, and Rey Auditory Verbal Learning task assessment results (Van Loenhoud et al., 2019). The ADNI-EF reflects the level of executive ability and includes a composite z-score consisting of Category Fluency, the Trail-Making Test, Digit Span Backwards, Wechsler Adult Intelligence Scale–Revised Digit–Symbol Substitution, and 5 Clock Drawing items (Van Loenhoud et al., 2019). The MMSE score indicates the overall cognitive level and the CDR-SOB indicates the level of dementia.

### Apolipoprotein E

All study subjects were tested for the APOE genotype. A description of the AOPE sample collection and testing methods can be found.^2^ APOE is composed of three types of alleles, ε2/ε2, ε2/ε3, ε3/ε3, ε2/ε4, ε 3/ε4, and ε4/ε4 of different groups of subtype types. Patients were divided into two groups, whereby those with the APOE ε2/ε2, APOE ε2/ε3, and APOE ε3/ε3 were classified as the APOE group that did not carry the ε4 allele (ε4- group), and those with the ε3/ε4 and ε4/ε4 were the APOE group that carried the ε4 allele (ε4 + group). In this study, those carrying the ε2/ε4 phenotype were excluded.

### Magnetic Resonance Imaging Acquisition and Processing

The MRI T1-weighted (T1-w) images analyzed in this study were obtained by downloading from the LONI image Data Archive. All images were acquired by Philips, Siemens, and GE scanners at baseline in the study subjects. MRI T1-weighted images in the ADNI database were standardized before uploading, and details of image scanning standards and processing methods can be found in the ADNI-MRI Technical Procedures Manual.^3^ All MRI T1-weighted images were analyzed using the AccuBrain^®^ V2.0 Magnetic Resonance Image Analysis System. AccuBrain^®^ is based on a multi-atlas non-rigid registration scheme for brain structure segmentation and quantification, which can automatically quantify 68 brain structure indicators. The accuracy of its hippocampal segmentation and quantification has been verified in multiple datasets (Abrigo et al., 2019). AccuBrain^®^ is a non-open source software and requires an application with BrainNow Medical Technology Limited for the relevant usage rights.

The AD-RAI is a comprehensive index based on the support vector machine model in AccuBrain^®^, and represents the similarity of an individual’s brain atrophy pattern with that in patients with AD. The value range is 0–1, whereby 1 represents a greater similarity. When the AD-RAI > 0.50, an individual is considered to have a brain structure that is more inclined toward AD and is considered more likely to have AD (Zhao et al., 2019; Mai et al., 2021). The AD-RAI status is considered positive when the AD-RAI > 0.5 (AD-RAI+) and negative when the AD-RAI ≤ 0.5 (AD-RAI-).

### Statistical Analysis

The MCI population at baseline was divided into four categories—the MCIs or MCIc group, and the APOE-ε4 or APOE-ε4+ group. Continuous variables of demographic and clinical characteristics were compared between the two groups using t-tests, and categorical variables were compared using the chi-square test. We also explored the relationships between the AD-RAI, APOE genotypes, and cognitive performance. To do this, we further divided the 733 participants into the four following groups: APOE-ε4+ plus AD-RAI-positive, only APOE-ε4+, only AD-RAI-positive, and both negative. Mann–Whitney U tests were used to compare the baseline cognitive measures among these groups. We further investigated the cross-sectional relationships using linear regression models, in which the interaction between APOE status and the AD-RAI levels was explored. A linear mixed-effects models with random intercept and slopes was used to investigate whether a higher AD-RAI and APOE4 genotype could predict faster cognitive decline of MCI patients. The interactions between AD-RAI, APOE genotype, and time were also tested.

We also investigated whether the AD-RAI and APOE genotypes could predict the rate of conversion from MCI to AD. Kaplan–Meier curves were used to show the survival rate and log-rank tests with Bonferroni correction were used to compare different curves. As we performed six times of comparisons (intergroup comparison of the four groups), *P* < 0.008(0.05/6) were thought to be statistically significant according to the Bonferroni method. Cox-proportional hazard regression models were used to test the association of the AD-RAI and APOE-ε4 on AD conversion. Analyses were performed in APOE-ε4+ and APOE-ε4- groups separately. Age, sex, and years of education were adjusted in these analyses, and hazard ratios (HRs) were reported. Statistical analyses were performed in R software (v. 4.1.0) and IBM SPSS Statistics (v. 25.0.0). Age, sex, and years of education were adjusted in all regression models. Statistical tests were two-tailed and *P* < 0.05 was considered significant. Bonferroni correction was conducted when making out multiple comparisons.

## Results

### Demographic Characteristics

This study included 426 patients in the MCIs group and 307 in the MCIc group at baseline stage. Demographic information is shown in Table 1. The MCIc group was older than the MCIs group (*P* = 0.042) and included a larger proportion of individuals carrying the APOE ε4 allele, but there were no statistical differences in gender or education between the two groups. The MMSE, CDR-SOB, ADNI-MEM, and ADNI-EF scores were statistically different between groups (*P* < 0.001). The AD-RAI was significantly higher in the MCIc group than in the MCIs group (*P* < 0.001).

**TABLE 1:** Demographic and general clinical characteristics by MCI, APOE groups.

	MCIs (n = 426)	MCIc (n = 307)	*P*_1_	APOE-ε4- (n = 364)	APOE-ε4+ (n = 369)	*P*_2_
Age (years), mean ± SD	72.66 ± 7.45	73.77 ± 7.00	0.042	73.59 ± 7.59	72.67 ± 6.94	0.088
Male, n (%)	244 (57.28)	189 (61.56)	0.244	215 (59.07)	218 (59.08)	0.529
Education (years), mean ± SD	15.84 ± 2.92	15.60 ± 2.74	0.760	15.97 ± 2.79	15.77 ± 2.89	0.760
APOE ε4, n (%)	175 (41.07)	113 (63.19)	<0.001	/	/	/
MCIc, n (%)	/	/	/	113 (31.04)	194 (52.57)	<0.001
MMSE, mean ± SD	27.92 ± 1.74	27.12 ± 1.72	<0.001	27.78 ± 1.71	27.39 ± 1.81	0.025
CDR-SOB, mean ± SD	1.31 ± 0.80	1.85 ± 0.93	<0.001	1.47 ± 0.89	1.62 ± 0.90	0.026
AD-MEM, mean ± SD	0.371 ± 0.645	–0.182 ± 0.528	<0.001	0.286 ± 0.677	–0.006 ± 0.605	<0.001
AD-EF, mean ± SD	0.372 ± 0.862	–0.109 ± 0.858	<0.001	0.248 ± 0.927	0.094 ± 0.850	<0.001
AD-RAI, mean ± SD	0.39 ± 0.34	0.64 ± 0.34	<0.001	0.45 ± 0.36	0.53 ± 0.37	0.003

*n, number; APOE ε4, apolipoprotein E ε4-allele; MMSE, Mini-mental State Examination; CDR-SOB, Clinical Dementia Rating Scale Sum of Boxes; AD-RAI, AD resemblance atrophy index; P_1_, MCIs vs MCIc; P_2_, APOE-ε4- vs APOE-ε4+.*

The 733 patients with MCI included 364 APOE-ε4- and 369 APOE-ε4+ patients. There were no significant differences between the APOE-ε4- and APOE-ε4+ groups in terms of age, sex, or education. In contrast, the APOE-ε4+ group had lower cognitive levels and higher AD-RAI values than the APOE-ε4- group (*P* < 0.05). The results are shown in Table 1.

For all 733 patients with MCI, we collected longitudinal cognitive data, and the number of cognitive measure visits was based on participant history taking or diagnostic visits, ranging from 1 to 20 visits per participant, with a median of 5. Details on the number of participants with longitudinal data of cognitive measures and diagnosis are shown in Table 2.

**TABLE 2:** Number of participants with longitudinal cognitive measures.

Time-point (month)	Diagnosis	MMSE	CDR-SOB	ADNI-MEM	ADNI-EF
**0**	733	733	733	733	731
**6**	53	646	642	646	646
**12**	116	697	695	699	697
**18**	44	259	255	259	258
**24**	127	571	578	575	571
**30**	3	5	6	5	5
**36**	121	458	462	469	462
**48**	0	302	307	322	315
**54**	3	4	3	6	6
**60**	39	192	196	212	60
**66**	3	16	17	24	23
**72**	23	134	150	165	160
**78**	4	17	20	30	30
**84**	19	100	120	135	131
**90**	3	17	19	33	32
**96**	27	91	111	102	100
**102**	0	29	27	26	26
**108**	0	75	74	57	56
**114**	8	11	11	9	9
**120**	15	37	37	28	27
**126**	3	9	9	7	7
**132**	6	18	18	12	11
**144**	2	11	12	11	11
**150**	3	5	5	5	5
**156**	1	3	3	2	2
**162**	0	3	3	0	0
**168**	0	1	1	1	1
**174**	2	1	1	2	2
**180**	1	1	1	0	0
**192**	0	2	2	0	0

### The Baseline AD-RAI and APOE-ε4 Status Were Associated Cross-Sectionally With Cognitive Performance

**FIGURE 1:**
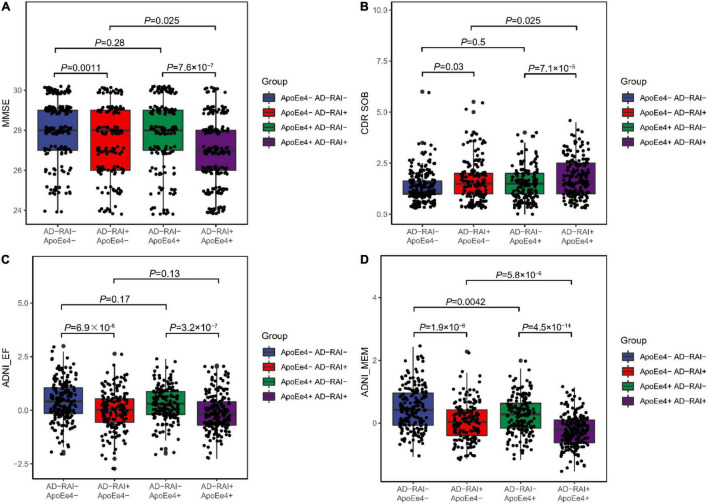
Associations of AD-RAI and APOE ε4 with cognitive measures among MCI patients. We categorized the participants into four groups: APOE-ε4+ plus AD-RAI-positive group (AD-RAI + APOE-ε4+), only APOE-ε4+ group (AD- RAI-, APOE-ε-4+), only AD-RAI-positive group (AD-RAI + APOE-ε4-) and both negative group (AD- RAI-, APOE-ε4-). We found that scores of MMSE **(A)**, ADNI-EF **(C)** and ADNI-MEM **(D)** were significantly higher in the two AD-RAI-positive groups. We also observed better memory performance **(D)** in the two APOE-ε4+ groups. The APOE-ε4+ plus AD-RAI-positive group performed better in CDR-SOB test than the only APOE-ε4+ group **(B)**. (PS: Jittering was used to avoid severe dot overlap).

We first compared the cognitive measures (MMSE, CDR-SOB, ADNI-EF, and ADNI-MEM) at baseline between the four groups (APOE-ε4+ plus AD-RAI-positive group, only APOE-ε4+ group, only AD-RAI-positive group, and both negative groups). The two AD-RAI-positive groups both had worse MMSE, ADNI-EF, and ADNI-MEM scores than the other two groups with the same ApoE-ε4 status as them. The APOE-ε4 + plus AD-RAI-positive group performed better in CDR-SOB test than the only APOE-ε4 + group. We also observed better memory performance in the two APOE-ε4- groups than in the other two groups (Figure 1).

We further tested the cross-sectional associations of the AD-RAI and APOE-ε4 status with cognitive measures using linear regression models (see Table 3). After adjusting for age, sex, and years of education, both the AD-RAI and APOE-ε4 status were associated with a lower MMSE score (APOE-ε4 status: β = -0.37, *P* = 0.003; AD-RAI: β = -0.76, *P* < 0.001), ADNI-EF score (APOE-ε4 status: β = -0.16, *P* = 0.008; AD-RAI: β = -0.39, *P* < 0.001), and ADNI-MEM score (APOE-ε4 status: β = -0.19, *P* = 0.023; AD-RAI: β = -0.42, *P* < 0.001). However, no significant cross-sectional association was found between the APOE-ε4 status and CDR-SOB score (β = 0.10, *P* = 0.112), while AD-RAI status was associated with CDR-SOB scores (β = 0.33, *P* < 0.001).

**TABLE 3:** Baseline associations of AD-RAI and APOE status with cognitive measures.

Independent Factors	Statistics	MMSE	CDR-SOB	ADNI-EF	ADNI-MEM
APOE Status, ε4 +	β	–0.37	0.10	–0.16	–0.27
	95% *CI*^1^	–0.62, -0.13	–0.02, 0.23	–0.28, -0.04	–0.36, -0.19
	*P* value	0.003*	0.112	0.008*	<0.001*
Baseline AD-RAI>0.5	β	–0.76	0.33	–0.39	–0.42
	95% *CI*	–1.20, -0.36	0.20, 0.46	–0.51, -0.27	–0.51, -0.34
	*P* value	<0.001*	<0.001*	<0.001*	<0.001*

MMSE, Mini-Mental State Examination; CDR-SOB, Clinical Dementia Rating Scale Sum of Boxes; MEM, Memory Function; EF, Executive Function. *Statistically significant.

*All models were adjusted for age, gender and education. No significant interaction was found between AD-RAI and APOE status.*

### The Baseline AD-RAI and APOE-ε4 Status Predicted Faster Cognitive Decline

**FIGURE 2:**
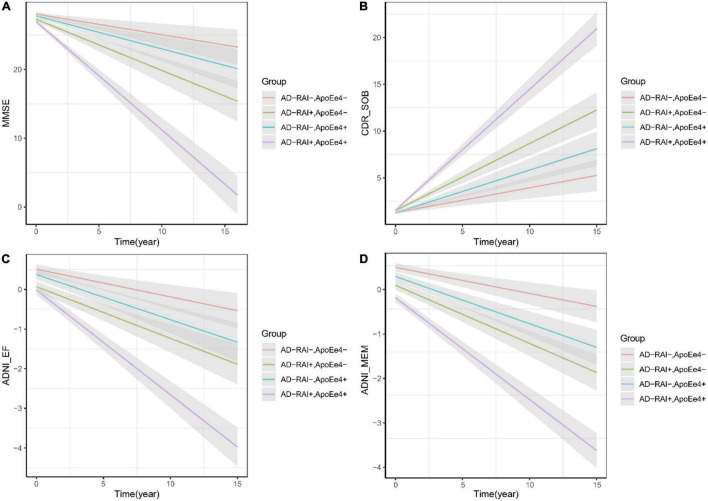
AD-RAI and APOE-ε4 as predictors of cognitive decline of MCI patients. Both AD-RAI-positive groups and APOE-ε4 + groups showed faster decline of memory **(D)** and executive ability **(C)** than their corresponding control groups. In our analysis, APOE-ε4+ failed to predict a faster decline of MMSE scores **(A)** among MCI patients with AD-RAI < 0.5 at baseline, but it was found to further amplify the acceleration of cognitive decline **(A–D)** in those with AD-RAI > 0.5.

Linear mixed-effects analysis showed that both APOE4 genotype and a baseline AD-RAI > 0.5 could predict faster yearly decline of memory (APOE-ε4 status: *P* = 0.005; AD-RAI: *P* < 0.001) and executive ability (APOE-ε4 status: *P* = 0.042; AD-RAI: *P* = 0.007) and a faster increase of dementia severity (APOE-ε4 status: *P* = 0.029; AD-RAI: *P* < 0.001) over 15 years. We did not observe any significant association between APOE4 genotype and the yearly rate of decline of MMSE scores among participants with an AD-RAI < 0.5 at baseline (*P* = 0.143). Interestingly, we also found that the APOE4 genotype could further amplify the acceleration of cognitive decline caused by the higher level of AD-RAI. The predictive ability of the AD-RAI and APOE remained significant over 10 years (Table 4 and Figure 2).

**TABLE 4:** Estimates of mean yearly rates of change in the cognitive measures of individuals with different APOE genotypes and AD-RAI levels.

Groups	MMSE	CDR-SOB	ADNI-EF	ADNI-MEM
APOE-ε4-AD-RAI-negative	–0.49	0.15	–0.06	–0.04
APOE-ε4-AD-RAI-positive	–0.70*	0.41*	–0.08*	–0.07*
APOE-ε4 + AD-RAI-negative	–0.64	0.38*	–0.10*	–0.08*
APOE-ε4 + AD-RAI-positive	–1.40*	1.00*	–0.15*	–0.12*

*MMSE, Mini-Mental State Examination; CDR-SOB, Clinical Dementia Rating Scale Sum of Boxes; MEM, Memory Function; EF: Executive Function.*

*Statistically significant when compared with the APOE-ε4- plus AD-RAI-negative group.

*The analyses were based on 15-year follow-up data and 10-year follow-up data respectively. There were no significant changes in the results. All models were adjusted for age, gender and education.*

### The Baseline AD-RAI Predicted the Conversion From MCI to AD in Both APOE ε4+ and APOE ε4- Individuals

**FIGURE 3:**
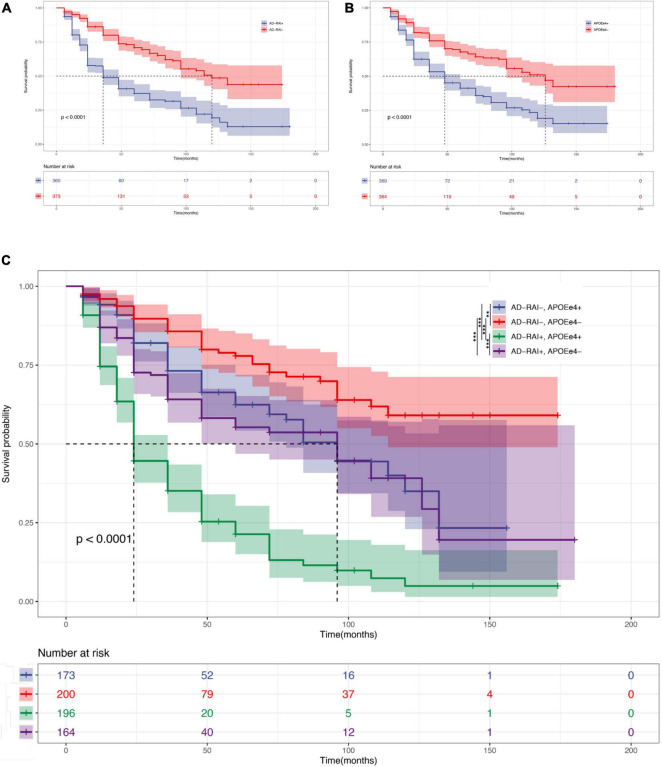
AD-RAI and APOE-ε4 as predictors in MCI. Kaplan–Meier curves showing that AD-RAI+ **(A)** and APOE-ε4+ **(B)** increased the risk of MCI progression. AD-RAI+ together with APOE-ε4+ reached the highest speed of AD conversion **(C)**. *P < 0.05; **P < 0.01; ***P < 0.001.

This analysis included 733 patients with MCI for whom we had APOE-ε4 test results, the baseline AD-RAI, and longitude diagnosis information for up to 180 months. Kaplan–Meier curves (see Figure 3) showed that an AD-RAI > 0.5 (median survival time = 36 months) and an APOE-ε4 + status (median survival time = 48 months) increased the risk of AD conversion. An AD-RAI > 0.5 was associated with an increased risk of AD conversion in both the APOE-ε4 + group (AD-RAI > 0.5, median survival time = 24 months; AD-RAI < 0.5, median survival time = 96 months; *P* < 0.001) and APOE-ε4- group (AD-RAI > 0.5, median survival time = 96 months; AD-RAI < 0.5, median survival time > 180 months; *P* < 0.001). A Cox-proportional hazard regression model showed, after correcting for age, sex, and years of education, that the AD-RAI + (*HR* = 2.82, 95% confidence interval, 2.22–3.58, *P* < 0.001) and APOE-ε4 + (*HR* = 2.31, 95% confidence interval, 1.83–2.92, *P* < 0.001) were still associated with an increased risk of MCI to AD conversion, and there was no interaction between them.

## Discussion

This study used data from a multicenter prospective cohort study to examine whether the AD-RAI can predict the risk of progression to AD and the level of cognitive decline in patients with MCI with APOE-ε4. We found that the AD-RAI was higher in the MCI to AD conversion population, that AD-RAI predicted the progression from MCI to AD, and that it was more accurate in predicting progression to AD in patients with MCI carrying APOE-ε4. The AD-RAI predicted cognitive decline and was strongly correlated with cognitive level.

While the effect of APOE-ε4 on cognitive alterations varies according to age, APOE has been reported to significantly affect the rate of memory decline, especially in situational memory, during midlife and early old age (Caselli et al., 2009; Rawle et al., 2018). On analyzing cognitive levels in patients with MCI carrying the ε4 allele and those not carrying the ε4 allele, we found a significant correlation between the AD-RAI and cognitive decline, and a longitudinal study revealed that higher values were associated with faster rates of cognitive decline and a synergistic effect with APOEε4. Cognitive functions such as learning and memory are dependent on hippocampal synaptic plasticity, constant neurogenesis, and communication within the brain’s memory network (Anacker and Hen, 2017). APOEε4 has been found to damage cognition-related brain structures and cognition-related pathways, increase tau deposition in medial temporal lobe regions, and accelerate hippocampal atrophy, further leading to cognitive deficits (Cho et al., 2016). Cognition is strongly correlated with atrophy of brain structures, and learning and memory impairments have been associated with atrophy of brain structures such as the hippocampus and amygdala (Yavuz et al., 2007; Sluimer et al., 2008; Guzmán-Vélez et al., 2016). Cognitive decline, such as memory decline, is common in patients with AD, and 90% of amnestic MCI cases progress to AD (Petersen et al., 2005; Hodson, 2018). The AD-RAI is strongly correlated with the composite memory score of the ADNI-MEM (Crane et al., 2012), and the AD-RAI is a composite of characteristic atrophied brain structures associated with AD (Zhao et al., 2019; Mai et al., 2021). Therefore, the AD-RAI can be used as a composite of indexes of cognitive-related brain structures underpinning functions such as memory to predict cognitive decline associated with AD.

Hippocampal atrophy and hippocampal volume are an important markers for diagnosing AD with dementia or as a prognostic biomarker for predicting the transition from MCI to AD, and have been widely used for AD screening and clinical diagnosis (Jack et al., 2013a; Ruan et al., 2016). Hippocampal atrophy is a better predictor of progression in Aβ-positive MCI populations than CSF Aβ because there is a plateau in Aβ aggregation, whereas there is no plateau in the rate of hippocampal atrophy (Jack et al., 2010; Jack et al., 2013b). The amygdala, temporal lobe, and insula have also been used as early biomarkers to distinguish MCI from AD (Dubois et al., 2007; De Jong et al., 2008; Jack et al., 2013a). However, as a heterogeneous disease, there is heterogeneity in the atrophy of brain structures in AD (Poulakis et al., 2018). The AD-RAI is based on an atrophy of brain structures that is characteristic of AD in the whole brain, and is 92% accurate in differentiating healthy subjects from patients with AD, which is almost identical to the accuracy of CSF biomarkers (Mai et al., 2021). In this study, we found that the AD-RAI predicted the risk of progression to AD in those with MCI, and the time to conversion to AD in patients with MCI was significantly shorter when their AD-RAI was >0.5. Consistent with our previous study, the AD-RAI was able to assess and predict structural brain indicators of the progression from normal functioning to MCI, and from MCI to AD (Zhao et al., 2019).

In the present study, the AD-RAI was better able to predict the progression to AD in patients with MCI carrying the APOE-ε4 allele than in patients with MCI not carrying the APOE ε4-allele. Patients with MCI carrying APOE-ε4 converted to AD in a shorter time than those not carrying the allele. Many studies have shown that APOE-ε4 promotes Aβ deposition in the brain (including neocortical areas) and disrupts the cortico-hippocampal network, which indirectly causes hippocampal atrophy through cortical denervation, further affecting the rate of hippocampal volume loss and gray matter atrophy (Geroldi et al., 2000; Schuff et al., 2009; Hesse et al., 2019; Yan et al., 2020). In contrast, the APOE-ε4-positive MCI population already has significant pathological changes, such as structural brain atrophy or Aβ deposition, and the use of only single characteristic brain structures or CSF Aβ has been found to lead to a decreased sensitivity in predicting the progression of MCI to AD (Jack et al., 2010). In another study, the AD-RAI was more accurate than single hippocampal and temporal brain structure characteristics in predicting and diagnosing AD (Mai et al., 2021). We performed a Cox-proportional risk analysis of the AD-RAI and APOE ε4, and found that the AD-RAI can independently predict the conversion of MCI to AD, independent of APOE. The APOE-ε4 allele is not only one of the strongest risk factors for AD but it is also a risk factor for Lewy body dementia (Mirza et al., 2019). Our previous study of the AD-RAI in differentiating AD from frontotemporal dementia revealed that the AD-RAI was more suitable for differentiating AD from healthy individuals and less accurate in frontotemporal dementia in terms of diagnostic accuracy (Yu et al., 2021). Therefore, the AD-RAI may be targeted to predict MCI progression to AD, and can accurately predict progression in patients with MCI with the APOE-ε4 allele.

The present study has some limitations. First, the population targeted in this study was patients with MCI; while our findings indicate that the AD-RAI can predict the risk of MCI progression to AD in those with the APOE-ε4 allele, the prediction of the risk of MCI or AD in APOE-ε4-positive healthy population is unknown, and it is possible that the AD-RAI differently predicts the risk of progression and cognitive decline in healthy individuals. Second, we excluded those carrying the ε2/ε4 allele, given the protective role of the ε2 allele against AD and the lack of studies on the ε2/ε4 allele in AD. Therefore, future studies could include healthy participants and those carrying the ε2/ε4 allele to further confirm the ability of the AD-RAI to predict AD risk. Third, the present study was a cross-sectional study of the AD-RAI and did not explore longitudinal changes in the AD-RAI to assess its numerical stability in continuous testing. Finally, we only considered the conversion of MCI to AD, without considering MCI subtypes and biomarkers. Therefore, the next step could be to analyze longitudinal studies of the AD-RAI in different populations to examine the validity of the AD-RAI for clinical and community applications.

In conclusion, the AD-RAI can be used as a prognostic imaging marker in patients with MCI carrying the APOE-ε4 allele, and can accurately and effectively predict the risk of progression to AD in both patients with MCI carrying APOE-ε4 and those without APOE-ε4. The AD-RAI was strongly correlated with cognition and could predict cognitive decline. Therefore, this study supports the use of the AD-RAI as a non-invasive diagnostic and prognostic tool for AD; the AD-RAI can be objectively, economically, simply, and efficiently applied in the clinic or community to identify the onset of early AD.

## Data Availability Statement

The raw data supporting the conclusions of this article will be made available by the authors, without undue reservation.

## Ethics Statement

The studies involving human participants were reviewed and approved by the Ludwig-Maximilians Universität München institutional review board (IRB). The patients/participants provided their written informed consent to participate in this study.

## Author Contributions

YM, ZC, JX, and JL designed the study. YM, ZC, and JX analyzed data, composition of figures and drafted the manuscript. QY, JT, WF, and LZ collected the data. VM, LS, and YL contributed to the MRI acquisition and processing. SY and ML interpreted data for the study. WL and JL contributed to the study supervision and critical review of manuscript for intellectual content. All authors gave their final approval of the version to be published and agreed to be accountable for all aspects of the work.

## Conflict of Interest

LS is the director of BrainNow Medical Technology Limited. VM is the chief medical consultant of BrainNow Medical Technology Limited. YL and LZ are employed by BrainNow Medical Technology Limited. The remaining authors declare that the research was conducted in the absence of any commercial or financial relationships that could be construed as a potential conflict of interest.

## Publisher’s Note

All claims expressed in this article are solely those of the authors and do not necessarily represent those of their affiliated organizations, or those of the publisher, the editors and the reviewers. Any product that may be evaluated in this article, or claim that may be made by its manufacturer, is not guaranteed or endorsed by the publisher.

## Footnotes

1
http://adni.loni.usc.edu/methods/

2
http://adni.loni.usc.edu/methods/

3
http://adni.loni.usc.edu/methods/documents/MRIprotocols
